# In Vivo Imaging Markers for Prediction of Radiotherapy Response in Patients with Nasopharyngeal Carcinoma: RESOLVE DWI versus DKI

**DOI:** 10.1038/s41598-018-34072-9

**Published:** 2018-10-26

**Authors:** Wei-Yuan Huang, Meng-Meng Li, Shao-Min Lin, Feng Chen, Kai Yang, Xiao-Lei Zhu, Gang Wu, Jian-Jun Li

**Affiliations:** 1 Department of Radiology, Hainan General Hospital, Hainan, China; 2 Research and Education Department, Hainan General Hospital, Hainan, China; 3 Department of Radiotherapy, Hainan General Hospital, Hainan, China; 4 Siemens Healthcare, MR Scientific Marketing NE Asia, Beijing, China

## Abstract

In this prospective study, we compared the performance of readout segmentation of long variable echo trains of diffusion-weighted imaging (RESOLVE DWI) and diffusion kurtosis imaging (DKI) for the prediction of radiotherapy response in patients with nasopharyngeal carcinoma (NPC). Forty-one patients with NPC were evaluated. All patients underwent conventional MRI, RESOLVE DWI and DKI, before and after radiotherapy. All patients underwent conventional MRI every 3 months until 1 year after radiotherapy. The patients were divided into response group (RG; 36/41 patients) and no-response group (NRG; 5/41 patients) based on follow-up results. DKI (the mean of kurtosis coefficient, Kmean and the mean of diffusion coefficient, Dmean) and RESOLVE DWI (the minimum apparent diffusion coefficient, ADC_min_) parameters were calculated. Parameter values at the pre-treatment period, post-treatment period, and the percentage change between these 2 periods were obtained. All parameters differed between the RG and NRG groups except for the pretreatment Dmean and ADC_min_. Kmean-post was considered as an independent predictor of local control, with 87.5% sensitivity and 91.3% specificity (optimal threshold = 0.30, AUC: 0.924; 95% CI, 0.83–1.00). Kmean-post values of DKI have the potential to be used as imaging biomarkers for the early evaluation of treatment effects of radiotherapy on NPC.

## Introduction

Diffusion-weighted imaging (DWI) has been widely applied to head and neck tumor detection, staging, characterization, and treatment response prediction^1–5^. However, motion and magnetic-sensitive artifacts caused by susceptibility-based distortion near bones and air might compromise the results of conventional single-shot echo-planar imaging (ss-EPI) DWI in the nasopharynx. A readout segmentation of long variable echo trains (RESOLVE) DWI with a higher spatial resolution and less artifact susceptibility than ss-EPI DWI was a better functional technique^6,7^. Recent studies used non-Gaussian diffusion models for brain DWI^8–10^ and for other tissues, including the head and neck^11,12^. As a non-Gaussian diffusion model, diffusion kurtosis imaging (DKI) could better depict the complicated water diffusion behavior in living tissue DWI^12^. The mean of kurtosis coefficient (Kmean) and the mean of diffusion coefficient (Dmean) might provide more information on tissue heterogeneity, vascularity, and cellularity than the apparent diffusion coefficient (ADC)^13^. We hypothesized that RESOLVE DWI and DKI could be used to assess treatment response in nasopharyngeal carcinoma (NPC). The ability to predict the local outcome of a primary tumor may help improve treatment planning and provide useful information about the need for additional re-planned radiotherapy or chemotherapy. The present study compared the performance of RESOLVE DWI and DKI for the prediction of radiotherapy response in patients with NPC and explored valuable imaging markers.

## Results

### Treatment outcome

We successfully obtained DWI and DKI parameter maps for all 41 primary NPC tumors in both the pre-treatment and early post-treatment periods. Among the 41 patients, 5 (12.2%) were found to be local failures, while 36 (87.8%) belonged to the local control 1 year after the end of radiotherapy. The intraclass correlation coefficient (ICC) of the two radiologists’ measurements for ADC_min_ was 0.73 and the DKI was 0.81.

### Prediction of radiotherapy response

All the parameter data from the pre-treatment and the early post-treatment periods and the percentage changes between these 2 periods are presented in Table 1. There were no significant differences in T stage (Pearson chi-square value is 3.081, *P* value is 0.214), gender (Pearson chi-square value is 0.236, *P* value is 0.627) and age (Pearson chi-square value is 0.317, *P* value is 0.574) between the two groups.

**Table 1:** Parameters Between Respond Group (RG) and No-respond Group (NRG).

Parameter	RG	NRG	*P* value
ADC_min_-pre (*10^−3^m^2^/s)	0.66 ± 0.09	0.66 ± 0.09	0.947
Dmean-pre (*10^−3^m^2^/s)	1.49 ± 0.46	1.29 ± 0.26	0.219
Kmean-pre	0.57 ± 0.16	0.77 ± 0.14	0.010
ADC_min_-post (*10^−3^m^2^/s)	1.30 ± 0.20	1.05 ± 0.17	0.001
Dmean-post (*10^−3^m^2^/s)	1.96 ± 0.45	1.41 ± 0.25	0.001
Kmean-post	0.22 ± 0.66	0.38 ± 0.11	0.000
ADCmin-change (%)	98.74 ± 37.13	59.70 ± 24.02	0.013
Dmean-change (%)	37.77 ± 32.54	10.96 ± 16.20	0.012
Kmean-change (%)	61.40 ± 7.91	51.09 ± 6.20	0.005

The pre-treatment ADC_min_ of the response group (RG) were similar to the no-response group (NRG), and there was no significant difference (*P* = 0.947). The pre-treatment Dmean of the RG was larger than the NRG, but it was not significant (*P* = 0.219). The pre-treatment Kmean of the RG group was statistically lower than that of the NRG group (*P* = 0.01) (Table 1).

**Figure 1:**
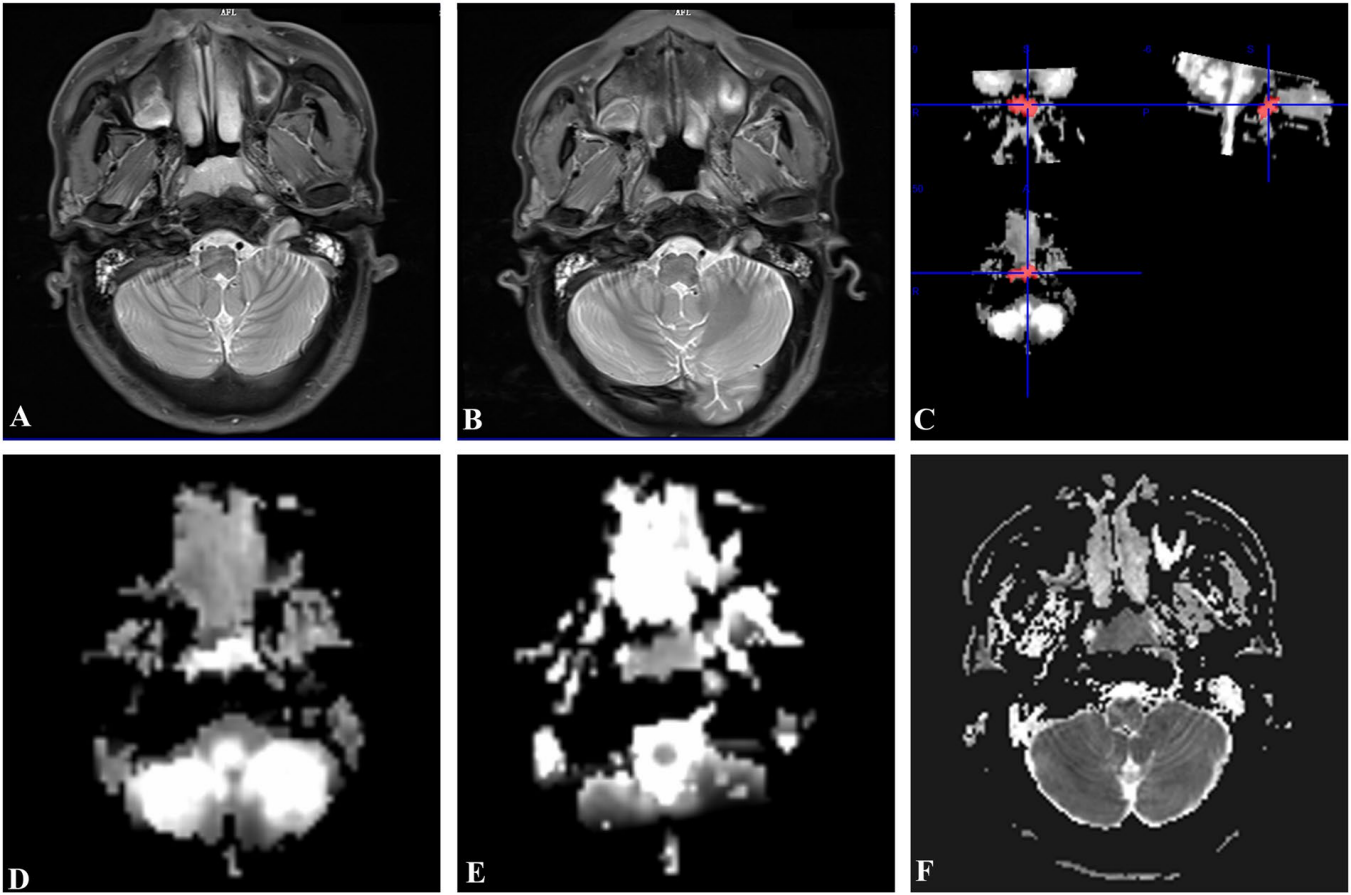
A 48-year-old man with NPC in RG. Pre-treatment PdWI (**A**) showed the lesion located at the bilateral mucous membrane of the nasopharynx. No residual tumor was detected on axis PdWI after radiotherapy (**B**). 3D-ROI were manual drawing including the whole lesion on Kmean map (**C**). The Dmean (**D**) and Kmean(**E**) values were 1.36 × 10^−3^ mm^2^/s and 0.7 before treatment, respectively. The ADC_min_ (**F**) was 726.1 × 10^−3^ mm^2^/s before treatment.

**Figure 2:**
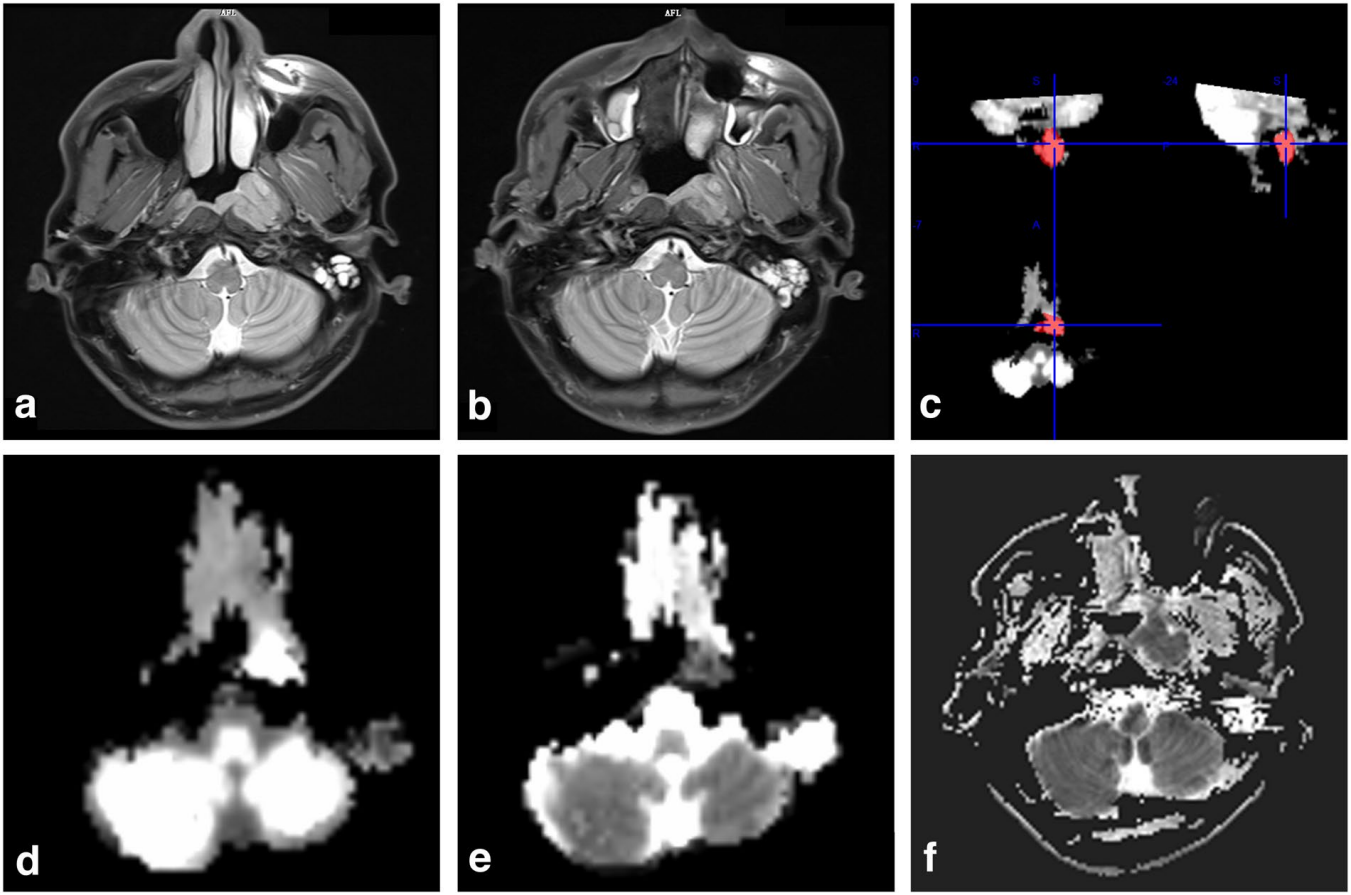
A 23-year-old man with NPC in NRG. Pre-treatment axis PdWI (**a**) showed lesions located at the left nasopharyngeal wall and cavum. Residual tumor was detected after radiotherapy (**b**). A manual drawing of an ROI within the boundaries of the NPC lesion on the Kmean map is also shown (**c**). The Dmean (**d**) and Kmean (**e**) values were 0.96 × 10^-3^ mm^2^/s and 0.95 before treatment, respectively. The ADC_min_ (**f**) was 754 × 10^-3^ mm^2^/s before treatment.

All parameter values at post-treatment and the percentage change of Kmean, Dmean, and ADC_min_ were statistically different between the groups (*P* < 0.05). Among these parameters, the Kmean-post, Kmean-change, ADC_min_-post, and ADC_min_-change were statistically significant. After radiotherapy, ADC_min_ value rose, Dmean rose, Kmean declined. These changes were more significant in RG than in NRG (*P* < 0.01) (Table 1). Figures 1 and 2 present a case example of the RG and the NRG. The Fig. 1 showed a NPC patient belong to RG. Pre-treatment proton density-weighted imaging (PdWI) (a) showed the lesion located at the bilateral mucous membrane of the nasopharynx. No residual tumor was detected on PdWI after radiotherapy (b). Region of interest (ROI) were manual drawing including the lesion on Kmean map (c). The Dmean (d) and Kmean(e) values were 1.36 × 10^−3^ mm^2^/s and 0.7 before treatment respectively. The ADC_min_ (f) was 726.1 × 10^−3^ mm^2^/s before treatment. The Fig. 2 showed a NPC patient belong to NRG. Pre-treatment PdWI (a) showed lesions at the left nasopharyngeal wall and cavum. Residual tumor was detected after radiotherapy (b). A manual drawing of an ROI on the Kmean map is also shown (c). The Dmean (d) and Kmean (e) values were 0.96 × 10^−3^ mm^2^/s and 0.95 before treatment, respectively. The ADC_min_ (f) was 754 × 10^−3^ mm^2^/s before treatment.

**Figure 3:**
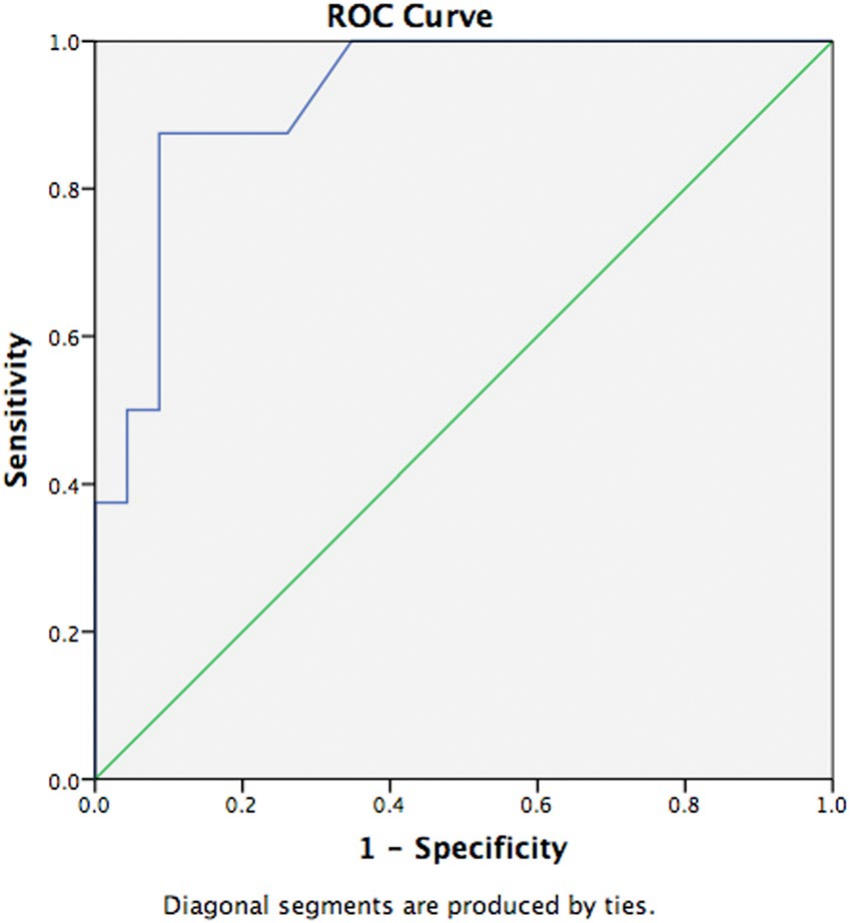
ROC curve of Kmean-post. The optimal Kmean-post threshold for distinguishing the RG from the NRG was 0.30, with 87.5% sensitivity and 91.3% specificity (AUC: 0.924; 95% CI, 0.83-1.00).

We chose Dmean-pre, Dmean-after, Kmean-pre, Kmean-after, ADC_min_-pre, ADC_min_-after, Dmean-change, Kmean-change, ADC_min_-change as factors for analysis by multivariate logistic regression models. The multivariate analysis revealed that the Kmean-post was considered an independent predictor for determining local control (Table 2). From the receiver operating characteristic (ROC) curve analysis, the optimal Kmean-post threshold for distinguishing the RG from the NRG was 0.30, with 87.5% sensitivity and 91.3% specificity (the area under the curve (AUC): 0.924; 95% confidence intervals (CI), 0.83–1.00) (Fig. 3).

**Table 2:** Significant parameters in the multivariate logistic regression models.

Parameter	Odds ratio	*P* value
Kmean-post	3.736E + 12 (14.210, 9.823E + 23)	0.031

Data are odds ratios and *P*-values. Data in parentheses are 95% confidence intervals.

## Discussion

Our preliminary study (patient cohort, number = 31) demonstrated that DKI could be a noninvasive tool to predict the early response to radiotherapy in NPC patients^14^. Then, based on our preliminary study, we continue to collect patients. This preliminary study investigated the ability of both DKI and RESOLVE DWI at 3 T to assess early treatment responses to radiotherapy in NPC patients. DKI and RESOLVE DWI parameters showed good reliability with high ICC values. Our findings revealed that all post-treatment and percentage changes of parameters between the pre-treatment and the early post-treatment period were statistically different between the groups, while the absolute values of pre-treatment ADC_min_ and Dmean were non-significant. Kmean-post was suggested to be the most powerful predictor of local control in NPC.

It remains controversial whether pre-treatment ADC values can predict radiotherapy response in NPC patients. Several studies have proposed clear correlation baseline ADC values with treatment response to NPCs^3,15^, while some showed more moderate results^12^. Our results did not find significant evidence indicating the predictive role of pre-treatment ADC_min_ and Dmean. Similar to our results, Chen *et al*.^16^. found there was no significant difference in pretreatment ADC between the RG and the NRG in stages III-IV NPCs after neoadjuvant chemotherapy. Hong *et al*.^17^. found no significant differences in pretreatment ADC between patients with and without residual tumors examined by MRI or biopsy 3 months after radiotherapy. However, Zhang *et al*.^3^. Suggested that pretreatment ADC was an independent prognostic factor for local control and disease-free survival. These controversial results might suggest that pre-treatment ADC values have wider fluctuation. Our results showed that the ADC_min_-post and the percentage change of ADC_min_ could predict the local control. Another study conducted by our team demonstrated that pre-therapeutic ADC_min_ failed to predict the primary central nervous system lymphoma outcomes, but ADC_min_ changes and percentage changes after one cycle of chemotherapy could more precisely predict treatment responses^18^. A possible explanation is that pre-therapeutic intratumoral cell density and stromal space cannot precisely reflect the treatment response. However, cellularity reduction caused by radiotherapy increases ADC values. Thus, post-therapeutic ADC growth might indicate the later tumor regression or decelerated growth and enable early detection of tumor response. Dmean showed a similar performance with ADC (Dmean-post and percentage change of Dmean had a significant difference). The possible reason was Dmean has a similar indication compared to ADC, indicating water diffusivity in tissues. However, Dmean derived from DKI contains specific information on the non-Gaussian diffusion behavior at ultrahigh b values, which may provide additional information from ADC^19^. Higher Dmean has been correlated with biophysical properties such as increased cellularity, vascularity, and infiltration^20^. Tumor cells sensitive to radiotherapy may show more morphology irregularities, wider intercellular spaces, and, therefore higher Dmean values. Early post-treatment parameter values may be more precise, indicating later tumor regression or decelerated growth and enabling the early detection of later tumor response. However, Dmean values showed greater variation than ADC_min_ values; the possible reason was that Dmean were more sensitive to the minute variations caused by therapy. Further study is needed to confirm its role in the prediction of therapy response.

In our study, the differences in Kmean-pre, Kmean-post, and the percentage change of Kmean were statistically significant. Kmean performed better than ADC_min_ and Dmean in predicting radiotherapy response in NPC. This is consistent with a rectal cancer study by Jing Yu *et al*.^21^. Several studies have demonstrated improved goodness of fit with a non-Gaussian model compared to the standard Gaussian model^22,23^. We found that the RG had a significantly lower pretreatment Kmean than the NRG. This might be explained by the fact that a higher Kmean is related to micro-necrotic areas and heterogeneity tissues in tumors due to the loss of cell membrane integrity. Tumor cells in these areas confront more hypoxic and acidic environments. Therefore, the effectiveness of radiation therapy is diminished^24^. Some studies have reported that Kmean is an effective parameter to identify the heterogeneity of cellularity and microstructural complexity in other tumors^25–27^. After radiotherapy, Kmean was reduced, and residual tumors showed significantly higher Kmean than non-residual tissues.

Moreover, all parameter values post-treatment and the percentage changes of Kmean, Dmean, and ADC_min_ showed statistically significant differences between the groups; only Kmean-post could be considered as an independent predictor for local control in multivariate analysis. A possible reason was that the percentage change of the parameters changed along with the pre-treatment and post-treatment parameter values, influencing the result of independent predictors. Kmean from DKI have the potential to be imaging markers for therapy surveillance and to guide treatment choices.

This study had some limitations. It was limited by a small sample size and a single institution. Larger studies may confirm the findings. Second, the number of patients was small, and thus we could not perform a subgroup analysis with divisions of histopathological differentiation status. Third, this study did not observe long-term treatment responses and overall survival. The next study will extend the follow-up period and exhaustively discuss the relationship between DKI techniques and radiotherapy effects.

## Materials and Methods

### Patient population

This study was performed at a single hospital from November 2014 until April 2017. All procedures were approved by the Medical and Health Research Ethics Committee of Hainan General Hospital. All methods were performed in accordance with the national guidelines and regulations. Each patient signed an informed consent form after the nature of the procedure had been fully explained. TNM status was determined according to the seventh edition of the American Joint Committee on Cancer (AJCC) staging system^28^.

Patients who were recently diagnosed with NPC, had not undergone treatment for NPC with a Karnofsky score >80, and had no contraindications to magnetic resonance imaging (MRI) scans were included. The exclusion criteria were patients with any other malignant tumors in the prior 5 years, those who failed systematic radiotherapy, and those who had distant metastasis. Forty-four patients met the inclusion criteria. Two did not start radiotherapy, and one discontinued due to personal reasons. Forty-one patients were analyzed and treated at Hainan General Hospital (Table 3). Staging was performed by MRI and computed tomography (CT) scans of the head and neck, ultrasound of the abdomen, CT scans of the thorax, and bone scans. Advanced stages were predominantly seen.

**Table 3:** Patient Characteristics.

No.	Sex	Age	AJCC T stage	RG/NRG
1	M	62	T3N2M0	NRG
2	M	23	T4N2M0	NRG
3	M	45	T3N2M0	NRG
4	F	53	T2N2M0	NRG
5	M	41	T4N3M0	NRG
6	M	56	T3N2M0	RG
7	M	48	T2N1M0	RG
8	F	57	T2N3M0	RG
9	M	66	T2N2M0	RG
10	M	57	T3N2M0	RG
11	M	41	T2N2M0	RG
12	M	65	T2N0M0	RG
13	M	63	T2N3M0	RG
14	M	65	T2N1M0	RG
15	F	67	T2N1M0	RG
16	F	62	T2N1M0	RG
17	M	57	T2N1M0	RG
18	M	76	T2N1M0	RG
19	F	45	T3N1M0	RG
20	M	20	T3N3M0	RG
21	M	30	T3N3M	RG
22	M	46	T3N1M0	RG
23	M	41	T3N2M0	RG
24	M	64	T3N3M0	RG
25	F	65	T4N2M0	RG
26	F	50	T2N1M0	RG
27	F	48	T3N3M0	RG
28	M	77	T2N2M0	RG
29	F	35	T3N2M0	RG
30	M	30	T4N3M0	RG
31	M	35	T4N2M0	RG
32	F	45	T3N1M0	RG
33	M	20	T3N3M0	RG
34	M	30	T3N3M0	RG
35	M	46	T3N1M0	RG
36	M	41	T3N2M0	RG
37	M	64	T3N3M0	RG
38	F	65	T4N2M0	RG
39	M	53	T2N2M0	RG
40	F	57	T3N3M0	RG
41	M	62	T2N3M0	RG

M: male, F: female; AJCC: American Joint Committee on Cancer; RG: Response group; NRG: No-response group.

### Treatment and response evaluation

Patients with NPC were treated with curative intent radiotherapy. Three-dimensional conformal intensity-modulated radiation therapy was obtained from the protocol for nasopharynx and neck radiotherapy. The total dose was 68.2–72.6 Gy divided into 31 to 33 fractions, and the total duration of radiotherapy was 43 to 54 days. Based on National comprehensive cancer network (NCCN) guidelines of NPC, patients with TNM stages over T2N1M0 undergo concurrent chemoradiotherapy. Chemotherapy precept is DPP (Cisplatin) 80–100 mg/m^2^ on the 1st, 22nd and 43rd days of radiotherapy.

The curative effect of radiotherapy was evaluated 1 year after the termination of radiotherapy. Patients were diagnosed with no residual tumors if MRI examination indicated no soft tissues in the nasopharynx or no local bulges due to thickening of the mucous membrane of the nasopharynx. Patients with residual tumors or local bulges found by MRI were examined by electronic nasopharyngoscopy (biopsy) under the guidance of imaging. Biopsy results confirmed all MRI findings. Patients with residual tumors were classified into the NRG or the RG.

### MR scan protocol

Patients underwent DKI and RESOLVE DWI MRI scanning before receiving their therapy (pre-treatment), and at an earlier stage of <48 h after radiotherapy (post-treatment). Patients underwent conventional MRI scanning before receiving their therapy (pre-treatment), at an earlier stage of <48 h after radiotherapy (post-treatment) and every 3 months until 1 year after radiotherapy. All patients were imaged using a 3 T clinical MRI scanner (Tim Trio; Siemens, Erlangen, Germany) with an 8-channel head and a neck array coil. MRI examinations included PdWI, RESOLVE DWI, and DKI sequences. Axial fast spin-echo PdWI was obtained using the short inversion time inversion recovery (STIR) technique (TR/TE, 5200/20 ms; FOV = 320 × 320 mm, slice thickness/gap = 4/1 mm, number of slices = 25, number of signal averages [NSA] = 2, and scan time = 2:04 min). Axial DWI was obtained using RESOLVE sequence (TR/TE, 5000/82 ms; section thickness/intersection gap, 5/0 mm; matrix size = 130 × 130; FOV = 320 × 320 mm; 3 directions; b value = 0 and 1000 s/mm^2^, and scan time = 10:57 min). A fat-suppressed single-shot spin-echo EPI sequence was used in the axial plane using 30 orthogonal diffusion directions for DKI examination (TR/TE = 8300/72 ms, FOV = 230 × 240 mm, slice thickness/gap = 4/1 mm, number of slices = 25, voxel size = 2.2 × 1.5 mm, reconstruction matrix = 224, IR delay = 240 ms, NSA = 2, SENSE factor = 3, water-fat shift = minimum, recon voxel size = 1.24 mm, b values = 0, 500, 1000, and 1500 sec/mm^2^, and scan time = 10:57 min).

### Post-processing and measurements

All MR images were analyzed independently by two experienced radiologists (W-Y.H. and F.C., with 9 years and 13 years of experience in clinical MR imaging, respectively) blinded to the patients’ clinical history and radiotherapy responses. All parameter values were averaged.

The ADC map was described by RESOLVE DWI using software provided by the manufacturer (syngo.via; Siemens). DKI datasets including corrected Dmean and Kmean were post-processed using the software tool diffusional kurtosis estimator (DKE, version 2.6, built on February 25, 2015) on an external workstation^15^. The ADC_min_ values and DKI parameters were measured using the software MRIcro (www.mricro.com, version 1.40, Chris Rorden, University of South Carolina, SC, USA) on an external workstation at the pre- and post-treatment periods. The freehand 3D ROI was drawn on the total volume of the primary lesions based on the b1500 maps using axial PdWI for reference. ROIs derived from the Kmean map were copied in the different parametric maps to ensure that the same areas were evaluated.

### Statistical analysis

Statistical analysis was performed using SPSS for MAC (version 22; IBM SPSS). The ICC of the parameter measurement between the two radiologists was processed. Differences in age, ADC_min_, Dmean, and Kmean between the RG and NRG were evaluated using the Mann-Whitney U test. Chi-sqare test was used to compare gender and T stage (T2, T3, T4) parameters between the 2 groups. RESOLVE DWI and DKI parameters were chosen for analysis by multivariate logistic regression models to determine whether they had independent predictive values with odds ratios (OR) and corresponding 95% CI. The detected predictive values were also assessed using ROC curves constructed for calculating the AUC. A *P* < 0.05 was used to infer statistical differences, while *P* < 0.01 indicated statistically significant differences.

## Conclusion

We found that DKI and RESOLVE DWI parameter values in the early post-treatment period and the percentage changes between pre- and post-treatment periods may predict radiotherapy responses in NPC. With improved detail and decreased image distortion, DKI showed promising results regarding response prediction after radiotherapy in NPC. We believe that DKI can be a robust high-resolution diffusion-weighted imaging technique at 3 T to assist contrast-enhanced nasopharynx MRI. Kmean have the potential to be imaging biomarkers for the early evaluation of treatment effects of radiotherapy in NPC.

## Data Availability

The datasets generated during and/or analysed during the current study are available from the corresponding author on reasonable request.
